# High-throughput solid microsampling through stochastic robotic automation

**DOI:** 10.1038/s42004-026-02153-w

**Published:** 2026-07-31

**Authors:** Adam Lisowski, Henryk Żołnowski, Keyan Villat, Alec Schmidt, Edy Mariano, Jean-Charles Cousty, Jérôme Guérin, Ashley Stark, Frédéric Vasserot, Achim Ammon, David Gueller, Florian de Nanteuil, Jean-Jacques Schwartz, Mathias Cherbuin, Marc Kunze, Giuseppe Tommaso Costanzo, Pascal Miéville

**Affiliations:** 1 School of Engineering and Management Vaud, HES‑SO University of Applied Sciences and Arts Western Switzerland, Yverdon-les-bains, Switzerland; 2 Swiss Cat+ West Hub, SB-ISIC, Ecole Polytechnique Fédérale de Lausanne EPFL, Lausanne, Switzerland; 3 Dietrich Engineering Consulting SA, Ecublens, Switzerland; 4 Chemspeed Technologies AG, Fuellinsdorf, BL Switzerland; 5 DSM-Firmenich, Meyrin, Switzerland

**Keywords:** Chemical engineering, Design, synthesis and processing

## Abstract

Solid sampling at the sub-milligram and milligram scale remains a major bottleneck for high-throughput chemistry or material sciences, as existing approaches rely on manual handling or slow deterministic microsampling that do not readily scale. Here we present STORMS, an automated STOchastic Robotic MicroSampling system that enables fast, reliable and parallel sampling of solid materials at sub-milligram and milligram masses. Rather than attempting deterministic mass control, STORMS leverages stochastic sampling combined with robotic automation, and glass encapsulation to achieve statistically robust and reproducible sample collection. We show that this approach delivers high precision and throughput across a range of solid materials, while supporting straightforward parallelization thanks to encapsulation and minimal operator intervention. Benchmarking against conventional sampling workflows demonstrates substantial gains in speed and reproducibility. By decoupling sampling reliability from deterministic mass control, STORMS establishes stochastic microsampling as a general and scalable strategy for solid analysis at the sub-milligram and milligram scale.

**Figure:**
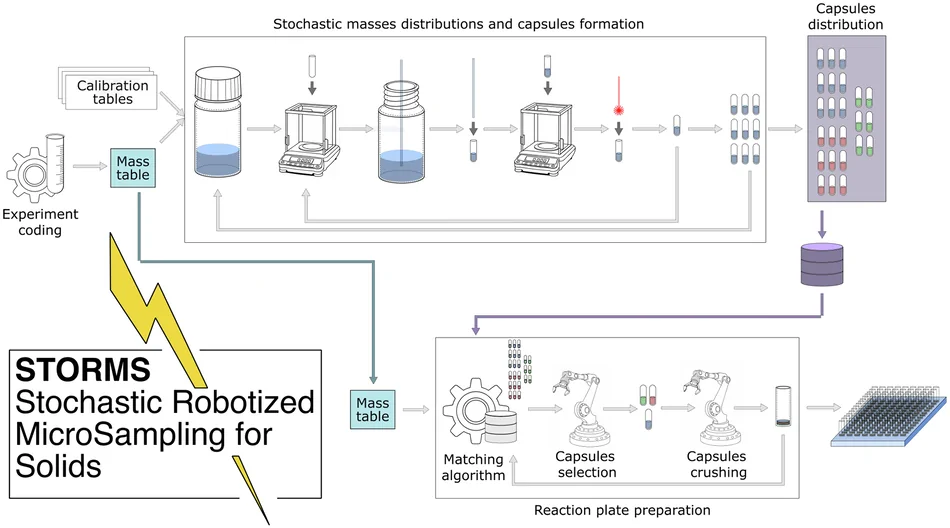

## Abstract (web summary)

High-throughput chemistry faces challenges in scaling solid sampling at sub-milligram and milligram levels due to manual and slow methods. Here, the authors introduce STORMS, an automated stochastic robotic microsampling system that enhances speed, precision, and reproducibility, offering a scalable solution for solid analysis with minimal operator intervention.

## Introduction

Self-driving laboratories have experienced intense development recently in the fields of pharmaceuticals and medicinal chemistry^1–3^, chemistry and catalysis^4–6^, and materials science^7–9^. Many of these developments occur at the level of generative algorithmics, proposing new molecules^10–12^, materials^13^, multiparametric optimization^14–17^, and synthesis pathways^18–20^, as well as automated lab equipment^21–24^. Others concern lab orchestration, equipment control, and data management^25–29^. Despite its crucial importance in all the previously cited domains, solid sampling, and more specifically sub-milligram and milligram scale sampling, remains sparsely studied. Currently, it relies on either human operators or commercial systems, such as the Chemspeed gravimetric dispensing units (GDU), which use a coring strategy to sample and distribute masses, or the Trajan Chronect/Mettler XPR, which uses grinders to dispense masses^30^. Some innovative studies discuss milligram-scale sampling methodologies that use robotic arms directly, such as a Biomimetic Solid Dispensing Robot^31^ and the FLIP^32^, which offer interesting prospects in terms of flexibility with respect to the complexity of the solids to be sampled.

Due to the previously described limited development, the existing human- and automated-based solutions have numerous limitations. These limitations include low throughput (several minutes per sample), limited versatility in terms of the types of solids that can be sampled in fully automated systems, and the risk of incompatibility-related failures. Conversely, human-operated systems are time-consuming, error-prone, and particularly tedious, especially for large arrays of reactions to be tested. The long time required for sampling (up to several hours for a 96-well reaction plate with multiple solids per well) in these existing methodologies also poses a high risk of sublimation, molecular alteration, and cross-contamination. Upon closer examination of existing technologies, the first coring approach (e.g., CST GDU) has two advantages: it does not have a dead volume related to the grinder, and it can work with any vial shape. However, this approach is highly sensitive to the properties of solids and powders, such as granularity, stickiness, and softness. In contrast, grinder-based dispensers (e.g., Chronect XPR) can accommodate a wider range of powders, as the grinding process intrinsically reduces particle size, allowing the powder’s granulometry to be adjusted to meet dispensing requirements, to some extent. They also better control potential powder flow during transfer, thereby limiting the risk of cross-contamination. However, the design of the grinding head creates a dead volume that can be problematic for rare or expensive substances, such as new drug candidates or new catalyst ligands. Additionally, each head must be associated with one specific chemical, which increases the cost. A campaign encompassing several hundred different chemicals, which is quite common in screening, will require a significant budget just for the grinders’ acquisition. Both grinders and coring tools are mostly inefficient with waxy solids or high-viscosity syrups. These substances are not technically solids, but they can be sampled as such.

Solids can be distributed in solutions using easy, fast, and precise liquid dispensing. However, this strategy is not always applicable, as it may require the use of an additional, potentially disruptive solvent to ensure sufficient solubility for homogeneous distribution of the substance. It may also be problematic to generate a solution for a limited number of test reactions, as there is a risk of massive loss, and small-scale solid sampling is still necessary. Another strategy is to support the target solids on an inert support, such as ChemBeads^33,34^, or deposit it on silica^35,36^. This strategy increases the average sampling size, which reduces experimental error. It also homogenizes and optimizes powder properties, improving reproducibility and efficiency. The deposition method also enables sampling of pseudo-solids, such as waxy substances and high-viscosity sirups, in a cleaner manner. On the other hand, these technologies introduce some uncertainty with respect to the amount of target chemical distributed for real during the process. This brings a stochastic dimension to the mass sampling that is already discussed by Tu et al.^33^. and that will be of prime importance in the approach we present here. The dissolution and deposition methods are of primary interest in drug discovery and medchem, but still require solid sampling, potentially in the milligram range (e.g., catalysts). They can be combined with efficient solid sampling methods to optimize them, but they do not fully replace them. This confirms the need for the development of efficient and versatile solid sampling methods.

**Fig. 1:**
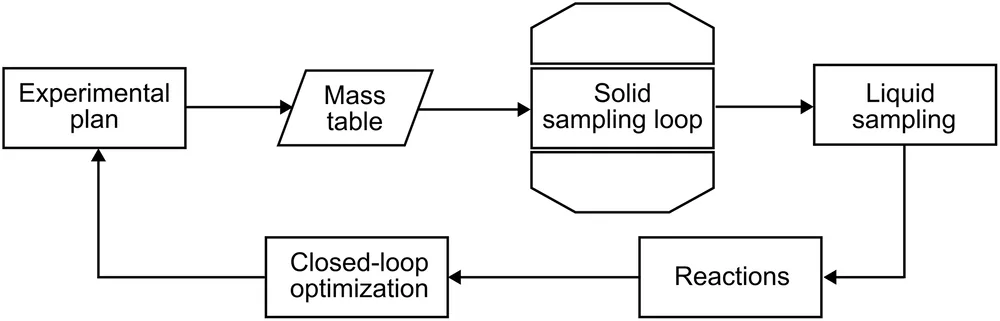
Deterministic approach, including masses calculation, sampling for a full synthesis workflow in an automated laboratory.

The main problem with existing solid sampling methods is their low throughput and inability to be parallelized. These limitations are strongly related to the deterministic approach used in mass generation. Target masses are fully defined by calculations in the context of reaction preparation, and samplers must cope with predefined values. Achieving precise masses requires the machine to have a feedback loop on the dispensing system, which suffers from delays and nonlinearities of the actuation and sensing components. These factors require reducing the dispensing speed, thus impacting the screening throughput. Since masses are only calculated at the reaction preparation stage, sampling cannot be disconnected from the reaction step, which limits parallelization, except at the cost of multiplying sampling equipment. These effects can be amplified in the case of a closed-loop optimization approach^5,14^ where multiple iterations require new calculations and samplings are required as visible in Fig. 1.

To overcome the limitations described above, we propose a stochastic solid-sampling strategy coupled with the generation of glass-sealed capsules. This approach aligns with the work of Zhong *et al.^37^ who highlighted the intrinsic stochasticity of chemical processes and reactions. By explicitly embracing this stochasticity, robotic platforms can operate in a less adaptive manner while naturally tolerating and exploiting small variations that would otherwise require substantial time and effort to control.

**Fig. 2:**
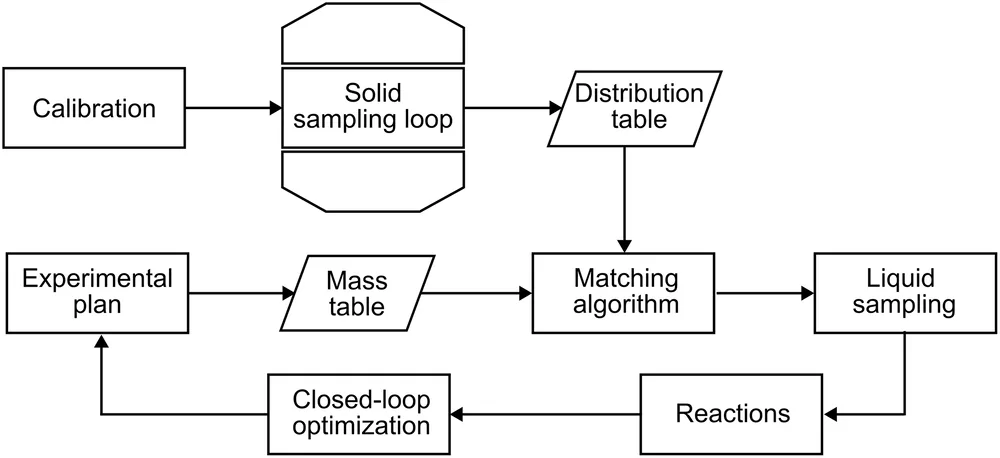
Stochastic masses generation and sampling for a full synthesis workflow in an automated laboratory. The gray tiles correspond to the stochastic process.

The underlying principle is the generation of a statistical distribution of accurately known masses through pre-calibration and rapid repetition of weighing operations. To be practically applicable, the stochastic solid-sampling approach must be combined with glass encapsulation, as the masses are prepared in advance of their use. Encapsulation, implemented using an adapted version of the Chemspeed SWILE system, enables the long-term storage, handling, and transfer of pre-weighed samples while preserving their integrity. This strategy decouples the sampling process from the synthesis step. This is achieved through the generation of a statistical distribution of precisely characterized, glass-encapsulated masses and an error-tolerant matching algorithm, as illustrated in Fig. 2.

Based on this idea, we will describe in this article the method named STORMS (STOchastic Robotized MicroSampling) aimed at generating a relevant yet stochastic, exactly known mass sample distribution that is individually laser-sealed in glass capsules. The inert sealed capsules are stored in a storage tower, from which they can be directly picked and used for further reactions. A combinatorial algorithm uses tunable parameters to best match the actual mass distribution with the specific synthesis or analysis batch mass table, picking the most appropriate capsules and delivering them to the correct reaction vessel. The solid is released via a capsule-breaking step directly on top of the reaction vessel. We will describe the detailed STORMS working principle, the hardware and software implementation and demonstrate its performance through experimental results in a range going from 0.1 to 20 milligrams for diverse types of solids.

### Storms

#### Stochastic microsampling principle

Automated milligram-scale solid dispensing, regardless of the sampling strategy (e.g., coring or grinding), traditionally relies on a feedback control loop between the dispensing tool and a 4–5 digit analytical balance to deliver a predefined mass. This deterministic approach systematically targets a fixed mass through adaptive control of the sampling tool. Using ISO 5725^38^ terminology, sampling accuracy (trueness and precision) depends on the compatibility of the dispensing tool with the material, the intrinsic properties of the solid (for example, grain size, which constrains resolution), and the balance’s readability and repeatability. The workflow is inherently time-intensive owing to iterative feedback and repeated weighing, and may still yield deviations from the target mass in the absence of integrated correction strategies.

**Fig. 3:**
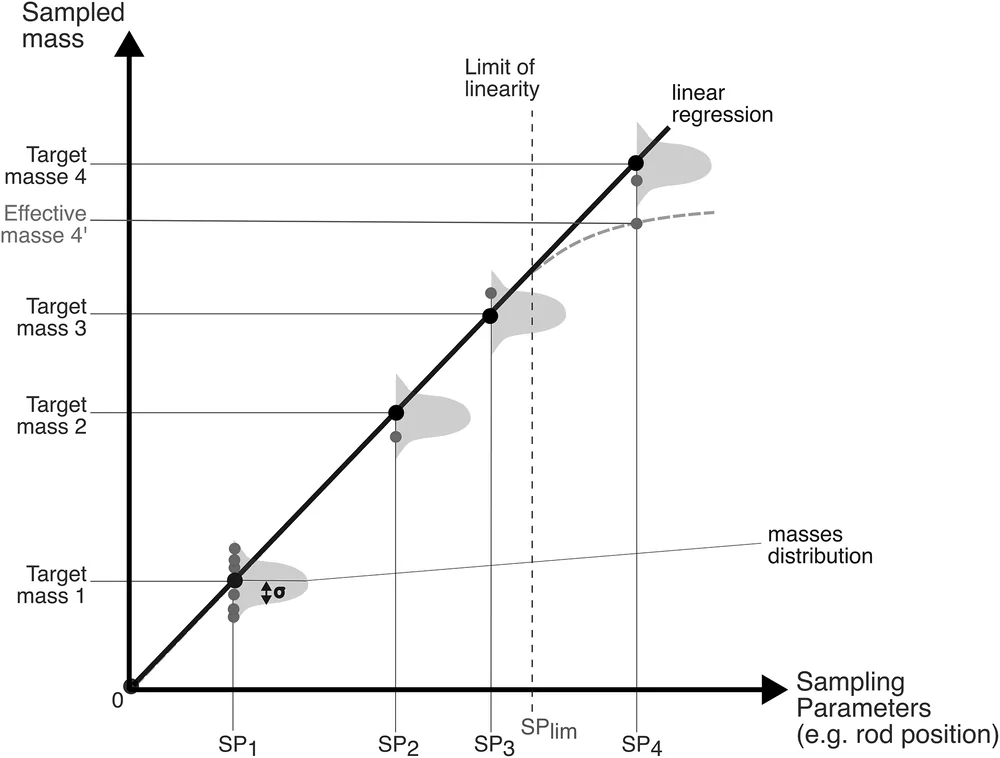
STORMS calibration workflow linking dispensed mass to sampling parameters. Black points represent target masses derived from linear propagation of the mass distribution measured at SP_1_. Gray points correspond to experimentally measured masses obtained at the respective SP_x_ values. Measurements at SP_1_ are used to determine the Gaussian dispersion parameter (σ) for the material–tool combination, while the full dataset establishes the linear regression model relating sampling parameters to delivered mass. When present, the onset of non-linearity defines the upper sampling limit (SP_lim_). Together, σ, the regression coefficients and SP_lim_ constitute the calibration dataset for a given material.

Automation excels at repetitive, predefined tasks with minimal adaptation and generally provides faster and more robust operation than adaptive robotic systems^39–41^. To reduce execution time, we eliminated feedback-loop control and intermediate weighing steps using a stochastic sampling strategy. We introduce STORMS (STOchastic Robotized MicroSampling), which relies on minimal pre-calibration of the sampling tool. Tool variability is incorporated into the chemical-space design through predefined tolerances and compensated during subsequent liquid handling (reagents, solutions, and solvents). The workflow requires only two weighings: an initial measurement of the empty receptacle (capsule) and a final measurement to determine the dispensed mass. The stochastic framework models dispensing using a Gaussian characterization of mass distributions (mean and standard deviation) combined with linear regression to relate target mass to sampling parameters for each solid (Fig. 3).

Calibration requires only a minimal set of measurements. An initial series of dispensing is used to determine the mean mass and standard deviation for a defined set of sampling parameters. The parameters are then varied linearly, and the corresponding masses are measured to establish a linear regression relating dispensing conditions to delivered mass. Across more than ten chemically diverse powders (see Results), the vacuum-assisted coring tool (see system design and operation) maintained a constant absolute standard deviation determined solely by the coring tube diameter over two mass ranges (0.1–1 mg and 1 to 8–10 mg). This invariance of dispersion enables a single Gaussian characterization of mass variability, after which the measured standard deviation can be propagated across the entire operating range. As a result, calibration requires substantially fewer experimental points.

As described above, the upper dispensing limit varies between ~8 and 10 mg depending on the tested material. During calibration, the linear operating range of the mass–parameter relationship is defined using the regression model (Fig. 3). The upper boundary of this range determines the material-specific sampling parameter limit (SP_lim_), corresponding to the onset of non-linearity and is associated with the highest mass possibly sampled in one shot for a specific material (m_max_). The couple [SP_lim_ ; m_max_], together with the regression coefficients and the Gaussian dispersion parameter (σ), constitutes the calibration dataset for a given solid. These parameters define the valid operating domain for stochastic dispensing and are explicitly incorporated into the sampling algorithm, ensuring that generated compositions remain within both physical (linearity-constrained) and statistical (τ-tolerant chemical space) boundaries.

The tolerance and correction strategy implemented in STORMS reframes solid dispensing from a mass-centric to a composition-centric paradigm. Historically, solids were *dispensed approximately but measured exactly*^42^ to a target mass and reagent or solvent volumes were subsequently adjusted to preserve stoichiometric ratios. STORMS formalizes this logic: controlled deviations in dispensed solid mass are accepted and composition is as much as possible restored through corrective liquid handling.

**Fig. 4:**
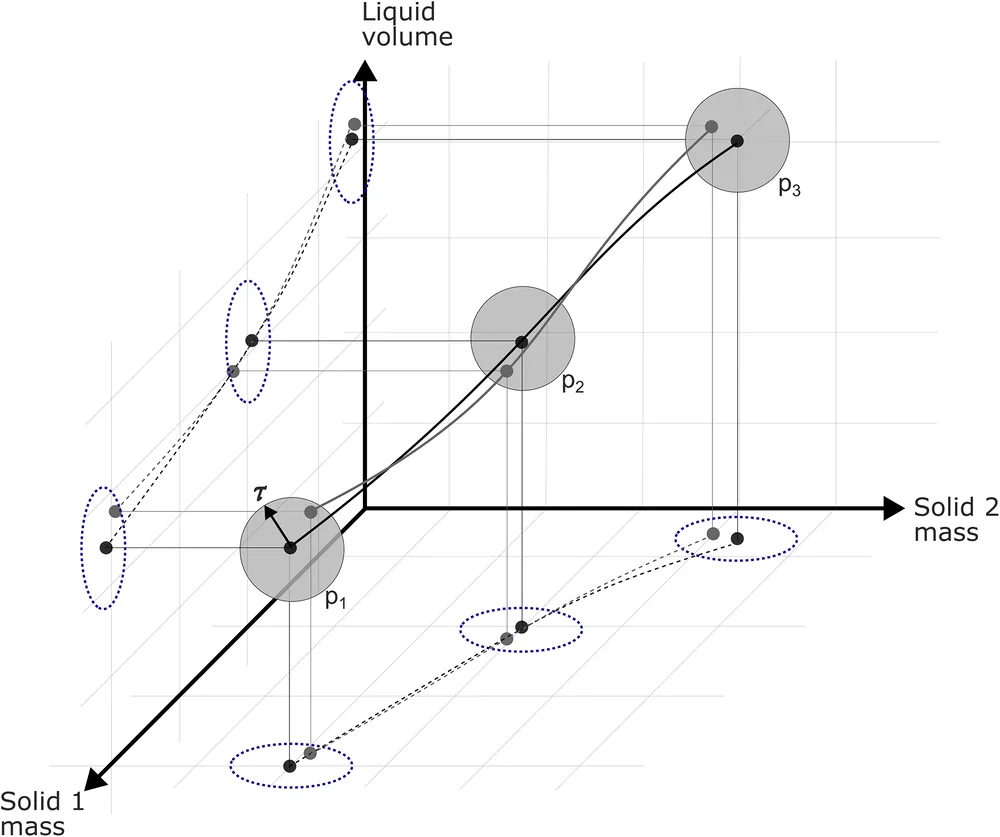
τ-tolerant stochastic sampling of a three-dimensional chemical space. Schematic representation of the STORMS sampling strategy in a three-dimensional reaction space (shown for clarity) defined by two solid masses (x–y plane) and one liquid volume (z axis), with an associated tolerance parameter (τ). Black points (pₓ) denote nominal design points defined by the experimental plan, whereas gray points indicate the effective compositions generated by stochastic dispensing. The shaded gray disks represent the τ-tolerance region surrounding each target point (dotted circles in planar projections, 3rd projection not shown for clarity). The nominal sampling trajectory is shown as a black curve, and the effective stochastic trajectory as a gray curve (dotted curve in the planar projections). The same principle generalizes to chemical spaces of arbitrary dimensionality.

Importantly, tolerance is introduced at a second level. Because the objective of high-throughput experimentation is to sample chemical space efficiently rather than to reproduce conventionally rounded or perfectly spaced design points, exact adherence to initially calculated compositions is not required. Instead, the goal is to generate a sufficient number of well-distributed experimental points with appropriate spacing to enable robust model fitting. STORMS therefore incorporates a tolerance parameter (τ) directly into the dimensions of chemical space, allowing deviations from nominal design points while preserving the statistical structure of the sampling strategy (Fig. 4).

For the STORMS chemical-space sampling strategy to enable efficient model fitting, the tolerance parameter (τ) must remain small relative to the inter-point distance in the experimental design (typically a few percent). This constraint ensures that stochastic deviations do not compromise the geometric structure required for regression or response-surface modeling. Accordingly, τ is defined dynamically as a function of the experimental design parameters. The resulting τ value is appended to the calibration dataset (including regression coefficients, σ and [SP_lim_;m_max_]) and incorporated into the recombination algorithm governing stochastic space sampling. The typical sampling time resulting from the stochastic approach is of 30 s / sampling, at least 4-5 x faster than the typical time required for sampling in deterministic mode.

#### Encapsulation

**Fig. 5:**
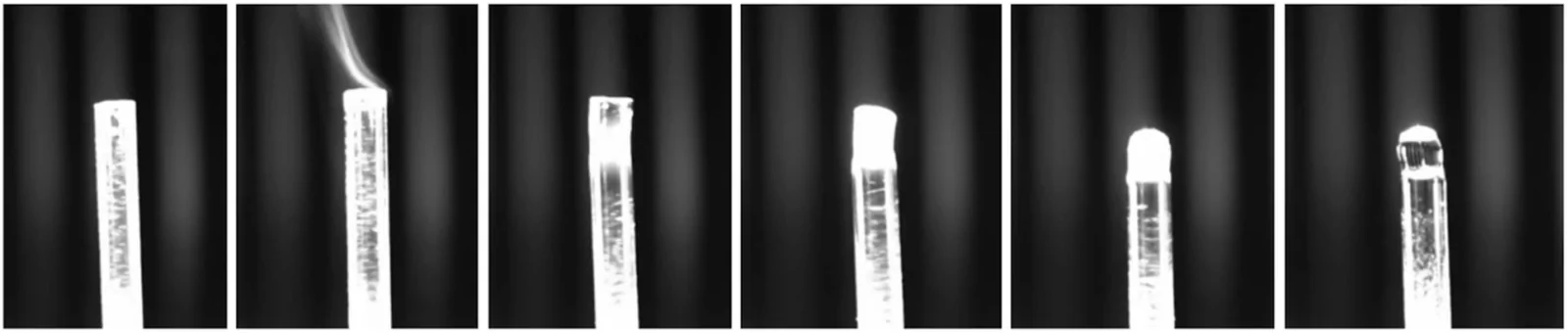
Sealing of the glass capillary tube via the utilization of an enclosed class IV 10.6 µm CO_2_ laser, forming the final capsule containing the solid to dispense.

A second acceleration is achieved through integration of a glass encapsulation step, based on the Chemspeed SWILE system, within the STORMS workflow (Fig. 5). On the contrary to a deterministic approach, in the stochastic approach the encapsulation is intrinsic to the process, as it enables accurate post-dispensing weighing of the solid and thereby fully decouples sampling from subsequent reaction preparation. After dispensing, solids are sealed in glass capsules using a 10.6 µm CO₂ laser and stored in a dedicated glovebox. Because sealing is fully parallelized with the sampling step, it does not increase overall process time. This modular strategy enables complete temporal and operational parallelization while providing enhanced protection for air-sensitive materials.

Together, the stochastic dispensing framework and glass encapsulation enable full parallelization of sampling operations. Sampling campaigns can be conducted independently of reaction setup in dedicated glovebox platforms (for example, Chemspeed Swing XL), and reaction plates (for example, Analytica Paradox) are subsequently assembled from pre-generated capsule libraries.

#### Matching algorithm and reaction plate population step

Reaction plates population with samples is controlled by a matching algorithm that integrates the target mass table defined by the experimental design, the calibration dataset (regression coefficients, σ and m_max_), the effective capsule distribution table and predefined sample combining rules to determine optimal capsule combinations. Implementation proceeds through a combinatorial capsule selection and dispensing (“crushing”) step, enabling automated, modular reaction plate construction. In closed-loop optimization campaigns involving multiple iterative cycles, an oversampling factor can be introduced during the initial sampling phase to generate a surplus of relevant capsules. This buffer enables successive reaction cycles to be executed without additional sampling, substantially reducing downtime and enabling continuous 24-h closed-loop optimization independent of operator intervention.

**Fig. 6:**
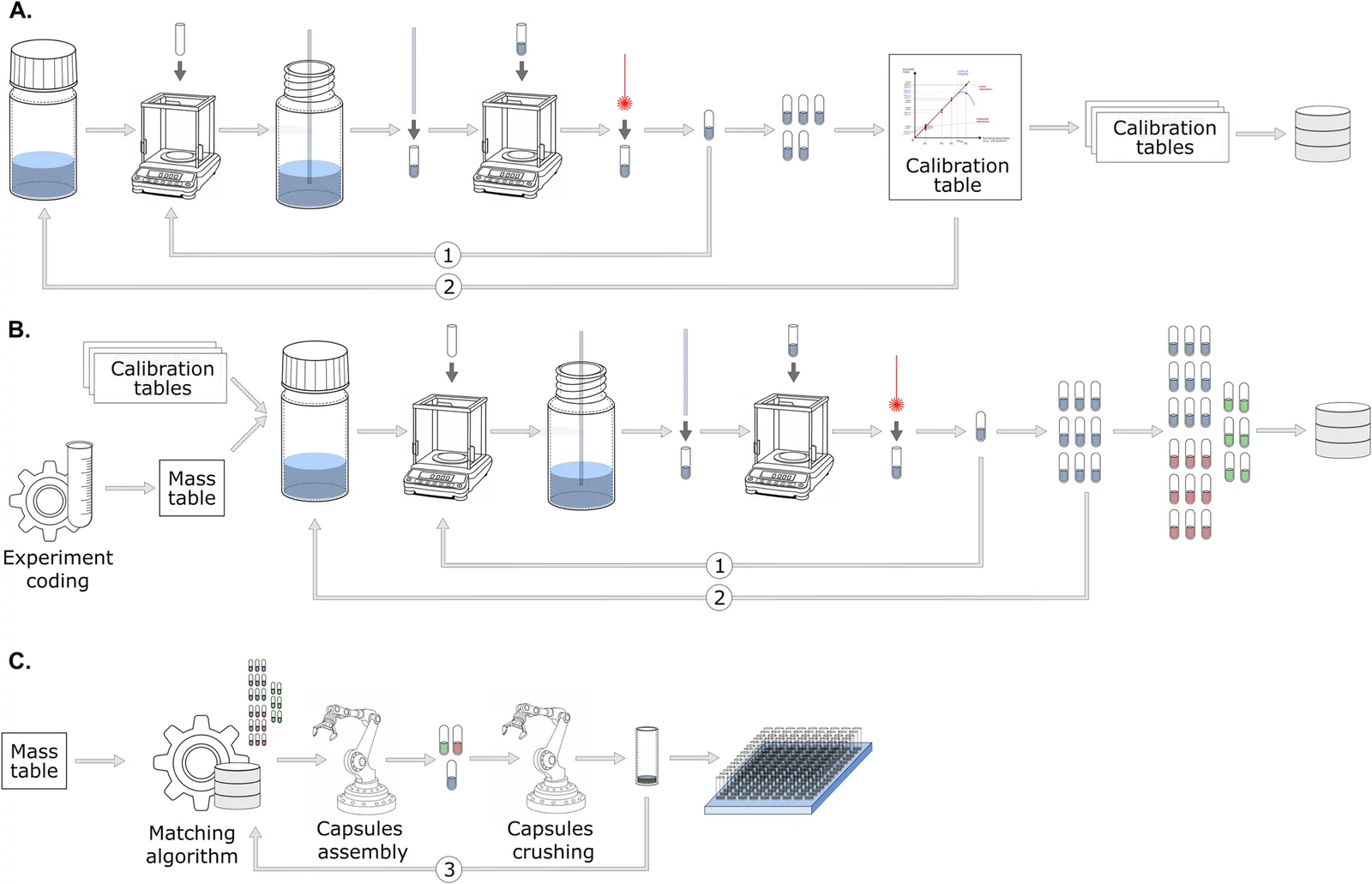
τ-integrated STORMS workflow for stochastic solid dispensing and closed-loop optimization. **A** Calibration. Minimal measurements establish the linear regression model relating sampling parameters to mass, the Gaussian dispersion parameter (σ) and the material-specific linearity limit (SP_lim_). Loop 1 iterates capsule generation; loop 2 iterates across solids, producing the calibration table. **B** Stochastic sampling and encapsulation. The calibration and experimental mass tables define τ-tolerant dispensing. Solids are dispensed, weighed, laser-sealed in glass capsules and stored. Loops 1 and 2 follow the same capsule and material iteration logic as in (**A**). **C** Reaction plate population with samples. A matching algorithm combines design constraints, calibration parameters and effective capsule distributions to select capsule combinations. Robotic pick-and-place and crushing are performed for each vial (loop 3) to generate the complete reaction plate. Oversampling during step B enables uninterrupted, fully parallelized 24-h closed-loop operation.

The plate population and capsule-crushing step is inherently a pick-and-place operation, a task well suited to robotic automation. At the Swiss Cat+ West Hub, using six-axis collaborative robots (Universal Robots UR3), this step requires ~30 s per capsule despite non-optimized hardware. Because capsule production is decoupled from reaction setup and performed in parallel during earlier campaigns, only the pick-and-place and crushing time determine effective reaction preparation throughput. This architecture results in a four- to fivefold acceleration compared with deterministic solid dispensing. For a 96-well plate containing three solids per vial (288 capsules), preparation time decreases from 9.6 h (2 min per dispensing step in a feedback-controlled deterministic workflow) to 2.4 h. In practical terms, this enables multiple full reaction plates to be assembled within a standard working day and supports continuous 24-h operation in closed-loop optimization campaigns. An additional advantage of the stochastic–encapsulation strategy is the reduction of cross-contamination risk. The short residence time during plate population minimizes exposure to sublimation and reduces the likelihood of material carryover from the sampling tool. Furthermore, glass residues generated during capsule crushing are negligible and do not interfere with the reaction, as experiments are conducted in glass vials. The complete process is summarized in Fig. 6. A detailed end-to-end workflow and a video, including all operational rules implemented at Swiss Cat + , is provided in the Supplementary Information.

### System design and operation

#### Vacuum-assisted coring module

**Fig. 7:**
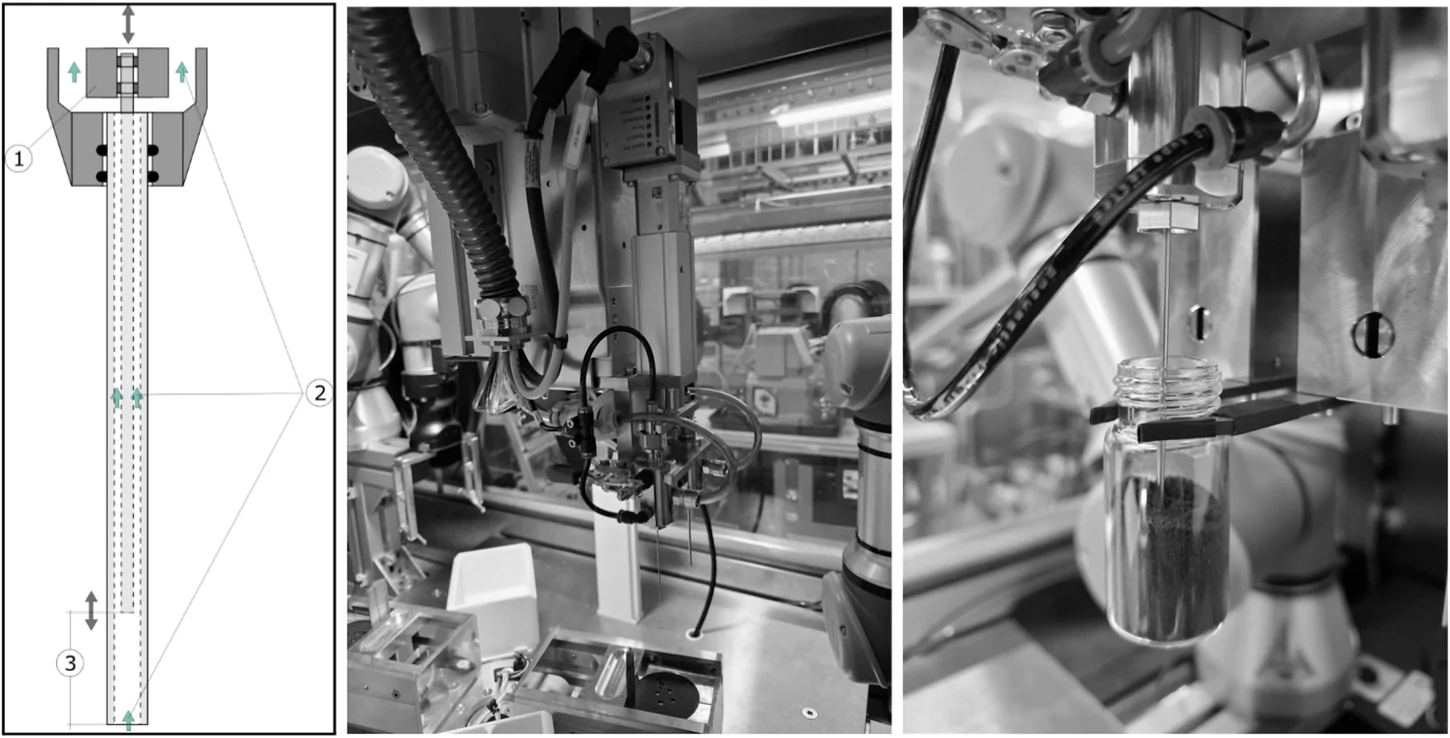
Vacuum-assisted solid coring module. Left, Schematic of the vacuum-assisted coring tool showing (1) the rod holder and vertical actuation module, (2) vacuum channels and (3) the sampling chamber, whose height is defined by the rod position and calibrated against the dispensed mass. Center, View of the overall static vacuum-assisted solid coring tool on top of the rotary platform used to deliver the coring tubes. Right, Photograph of the coring module integrated into the automated platform with a vial containing solid to be sampled positioned using a Universal Robot (UR3) with custom grippers.

Solid dispensing is performed using a dedicated vacuum-assisted coring tool (Fig. 7, *patent pending*). An external coring tube (inner diameter 0.6 mm for the 0.1 to 1 mg range, and 1.5 mm for the 1 to 10 mg range) penetrates the powder bed, while an adjustable internal rod defines the effective coring volume by setting the chamber height. The rod subsequently ejects the collected material directly into a glass capsule. The position of the vertical rod constitutes the primary calibration parameter governing the dispensed mass. To ensure robust operation across diverse materials, including fine powders and wax-like solids, vacuum is applied from the top of the tube up to the dosing chamber via a calibrated gap between the tube and the rod, to assist filling of the coring chamber. This configuration improves reproducibility and extends the accessible material scope.

The coring module remains static during operation. Vials containing solids and empty capsules are positioned beneath the tool using a six-axis robot (Universal Robots UR3 equipped with custom grippers). To minimize cross-contamination, stainless-steel coring tubes and rods are interchangeable and automatically inserted into the vacuum height-control head. A contamination-free capillary handling system was developed for automated exchange and adapted to both tube diameters. Two distributor units, mounted on a rotary platform together with waste compartments, service the static coring head.

#### Encapsulation and laser sealing

**Fig. 8:**
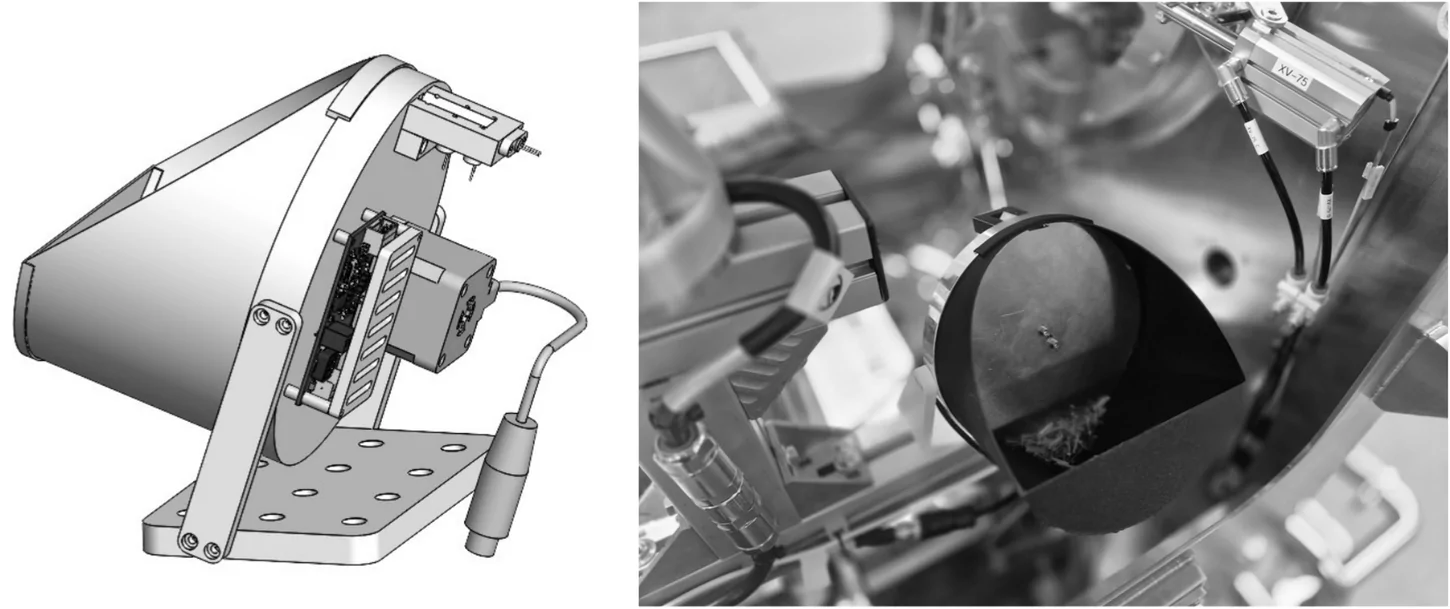
**Capsule dispenser Left**, Custom capsule dispensing system CAO, **Right**, Picture of the effective system mounted into the glove-box.

Empty glass capsules (outer diameter 2.8 mm, inner diameter 2.5 mm, and length 14 mm) are manufactured separately and must be distributed and correctly oriented for robotic handling during weighing (Fig. 6A, B) and subsequent filling using the vacuum-assisted coring tool. To enable reliable feeding and positioning, a dedicated capsule distributor was designed and integrated into the platform (Fig. 8).

**Fig. 9:**
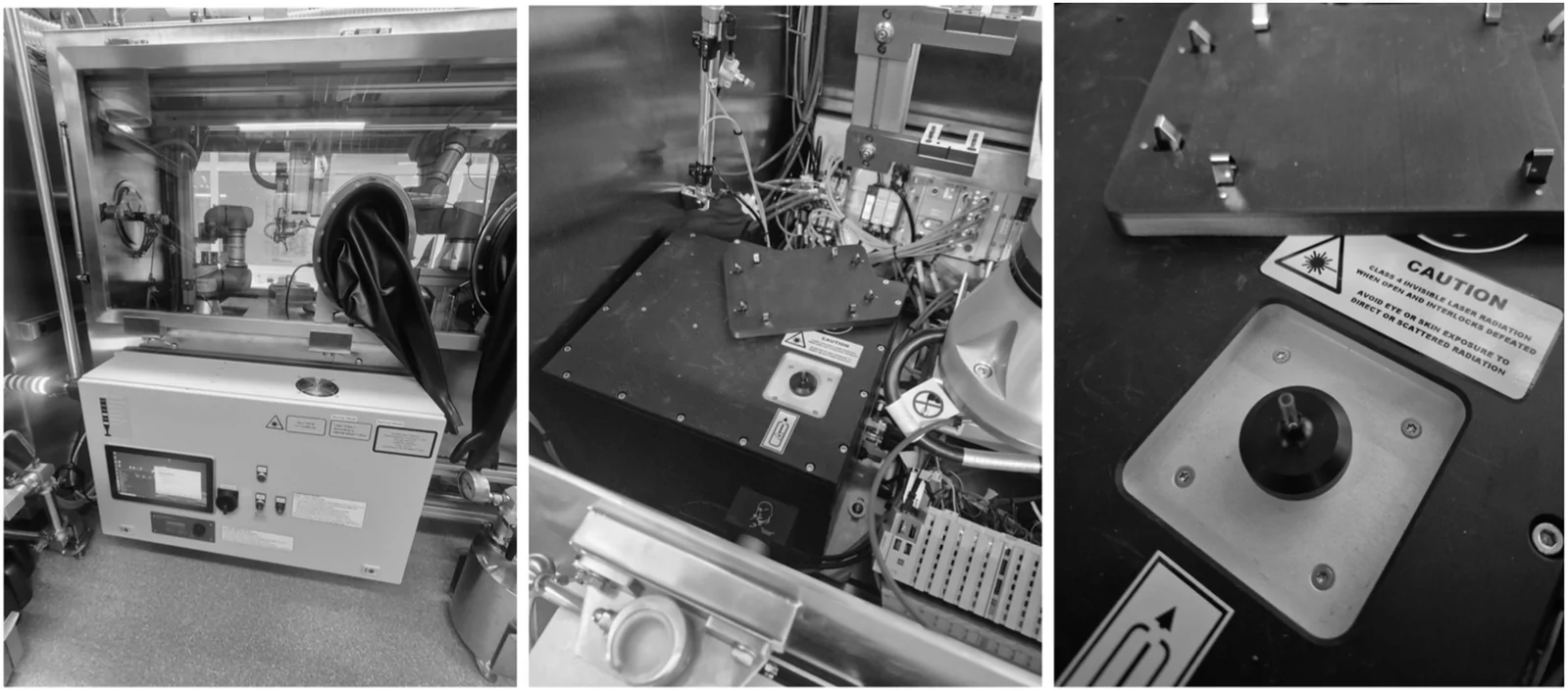
Enclosed laser-sealing system for capsules. Left, Class IV sealing laser external control box mounted on the glovebox walls. Center, Class IV laser internal enclosure showing the capsule loading/unloading zone (white rounded square). The internal enclosure confines the laser, enabling operation in an open environment. Right, Detailed view of the capsule positioned within the loading zone. During normal operation, the glass capsule is lowered into the enclosure and the aperture is sealed using a sliding lid.

After filling and post-dispensing weighing to determine the exact solid mass, capsules are sealed using an enclosed 10.6 µm CO₂ continuous-wave (CV) mode using Pulse With Modulation (PWM) class IV laser integrated within the glovebox. During sealing, capsules are maintained in a vertical orientation and rotated at 5 Hz to ensure homogeneous glass melting and uniform tube closure while preventing material contact with the sealing zone. The low thermal conductivity of glass limits heat transfer, enabling compatibility with thermally sensitive materials. The small capsule diameter (2.8 mm) provides high sealing reliability, with a success rate exceeding 99%. Incomplete seals are not critical, as capsules containing air-sensitive materials can be stored under inert atmosphere until use. The fully enclosed laser system allows safe operation in standard laboratory environments without dedicated laser infrastructure and can be handled by non-specialist personnel (Fig. 9).

#### Capsule pick and place and crushing

As outlined in Fig. 6C, capsule recombination is governed by a matching algorithm that integrates the experimental mass table, the effective capsule distribution table, and the material-specific calibration dataset, all stored in the laboratory database. A predefined set of rules and parameters selects (see supporting information S1) the optimal capsule combinations to deliver the required masses while satisfying the tolerance constraint (τ). A Rule-based decision algorithm is provided as Supplementary material (S2) and summarizes the parameter set and decision rules implemented at the Swiss Cat+ laboratory.

**Fig. 10:**
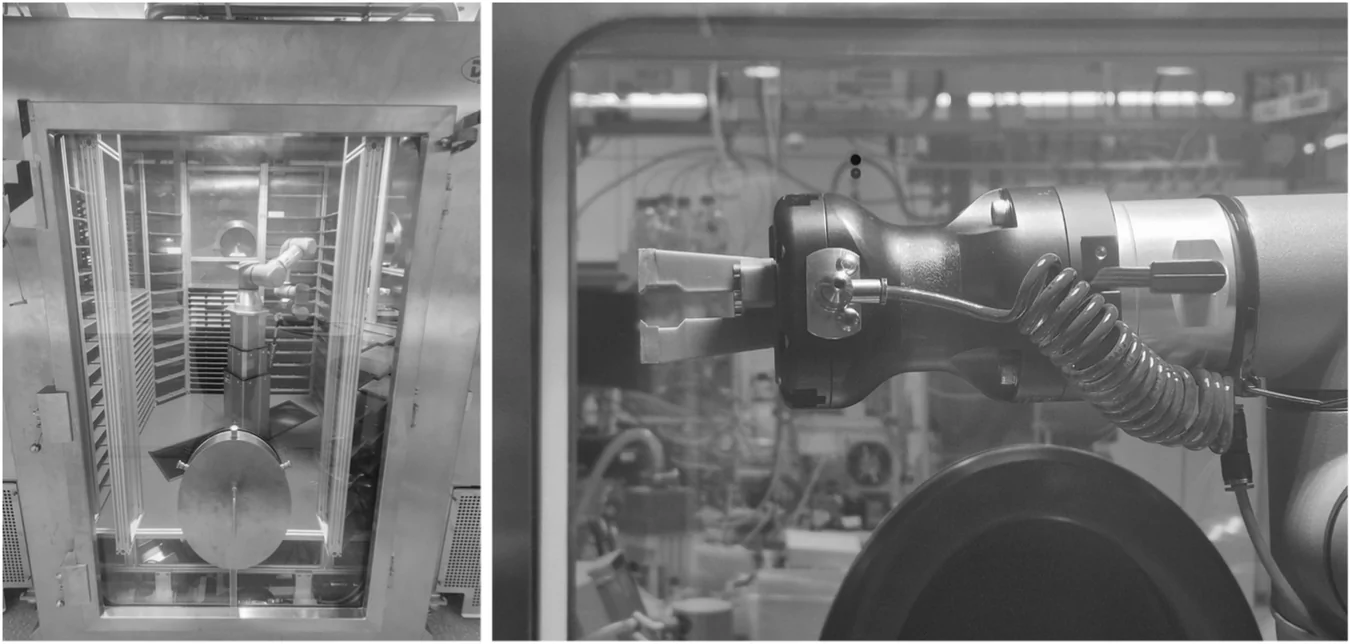
Capsule storage and robotic pick-and-place and crushing system. Left, Inert storage tower housing SBS-format 384-well plates containing sealed capsules and standardized 20-ml screw-cap vials for reagent supply. Right, Dual-function robotic end-effector comprising a pneumatic capsule gripper (front-facing) and a sharp pincer for lateral capsule crushing during reaction plate population.

Capsules are stored in SBS-format 384-well plates. Based on the matching table generated by the algorithm, the required storage plates are retrieved from the storage tower (Fig. 10, left) and positioned within the operating range of a dedicated six-axis robot (Universal Robots UR3). The robot is equipped with a dual-function end-effector comprising a pneumatic capsule gripper and a sharp pincer for lateral capsule crushing (Fig. 10, right). Selected capsules are transferred to a vertical holder to ensure correct orientation, then grasped by the pincer and positioned above the target reaction vial, with approximately 4 mm inserted into the vial. The capsule is crushed laterally, releasing the solid into the vial. The upper glass fragment is retained by the gripper and discarded into a dedicated waste container.

#### Performance validation

##### Accuracy and precision at sub-milligram and milligram scale

STORMS accuracy was calibrated and benchmarked using ten chemically and physically diverse powders (Table 1). The selected materials span a wide range of particle size distributions and flow characteristics, as quantified by Carr’s index, covering cohesive to free-flowing regimes. All compounds were chosen for low toxicity to enable systematic performance evaluation under controlled and safe operating conditions.

**Table 1:** Properties of powders used for STORMS validation

Products name	Size distribution in μm			Bulk density	Tapped density	Carrs Index
	10	50	90	$\rho B,{g}/l$	$\rho T,{g}/l$	${CI}=(\rho T\,-\,\rho B)/\rho T* 100,\, \%$
Granulac 70	16	107	213	710	910	22.0
Granulac 200	3	27	92	530	820	35.4
Tablettose 70	86	191	331	530	640	17.2
Flowlac 100	48	126	220	590	710	16.9
Spherolac 100	42	126	205	690	870	20.7
Inhalac 70	135	215	301	600	710	15.5
Inhalac 230	45	97	144	700	850	17.6
Inhalac 500	0	3	8	240	370	35.1
Sodium bicarbonate (NaHCO_3_)	-	-	-	1000	1200	16.7

Particle size distribution, bulk density, tapped density and corresponding Carr’s index (CI; flowability indicator) are reported for the 10 materials used to assess accuracy and repeatability. Particle size distribution was not available for the sodium bicarbonate batch.

**Fig. 11:**
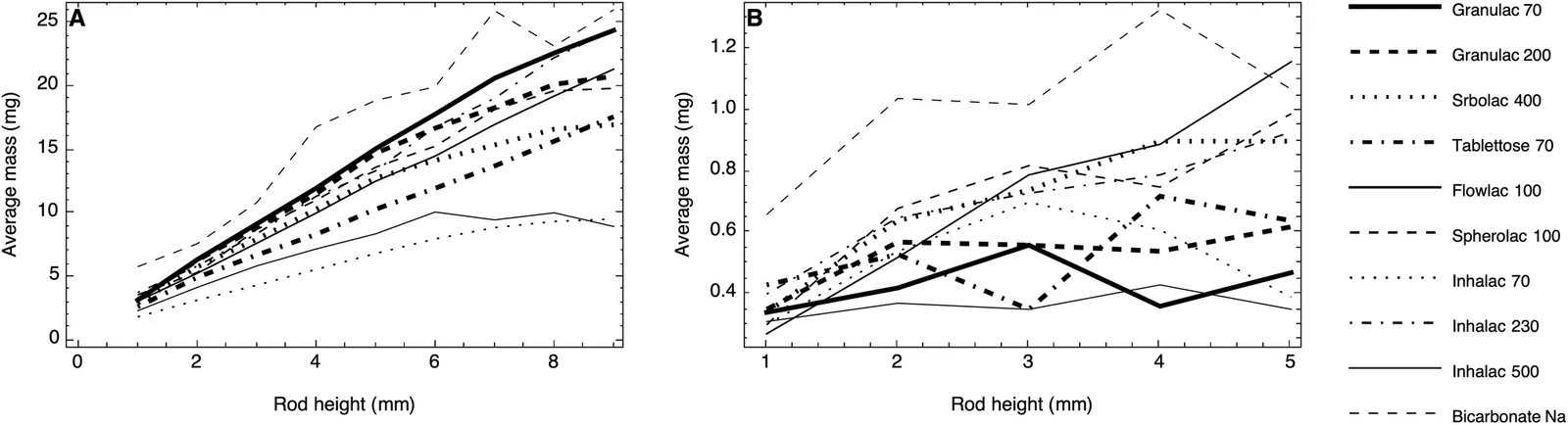
Calibration curves for vacuum-assisted coring tools of different diameters. **A** Average dispensed mass as a function of chamber height for the 1.5 mm inner -diameter coring tube across ten representative compounds (Table 1). Points represent mean values (*n* = 10). The average standard deviation (0.24 mg) is omitted for clarity. The linear relationship between mass and rod height defines the operating calibration range. Inhalac 500 and Spherolac 100 exhibit deviation from linearity above ~ 6 mg, consistent with the behavior described in Fig. 3. **B** Corresponding data for the 0.6 mm inner -diameter tube, with an average standard deviation of 0.06 mg. Deviations from linearity are more important at this scale. (data available in supplementary data D1 and D2).

In the sub-milligram regime, using the 0.6 mm inner-diameter coring tool, and in the milligram regime, using the 1.5 mm outer-diameter with 20 mm of powder inside the container, we obtain a good linearity between the rod height and the sampled mass on a majority of tested powders (Fig. 11).

Linearity and dispersion are improved in the milligram regime relative to the sub-milligram range, primarily because the absolute measurement uncertainty of the analytical balance (0.2 mg effective repeatability under operating conditions, ≤ 0.01 mg nominal according to vendor) contributes proportionally less to the total error at higher masses. In the sub-milligram domain, this fixed instrumental uncertainty represents a substantial fraction of the target mass, leading to increased relative error and reduced apparent linearity. Additional contributions arise from the geometry of the coring tool (tube inner diameter) and powder-specific properties, including particle size distribution. To minimize the impact of balance repeatability and readability on relative precision, the matching algorithm (S1, S2) preferentially targets masses in the 3–5 mg range whenever compatible with the experimental design. Sub-milligram dispensing remains accessible for chemical-space construction but is deployed selectively to maintain overall accuracy and statistical robustness.

**Fig. 12:**
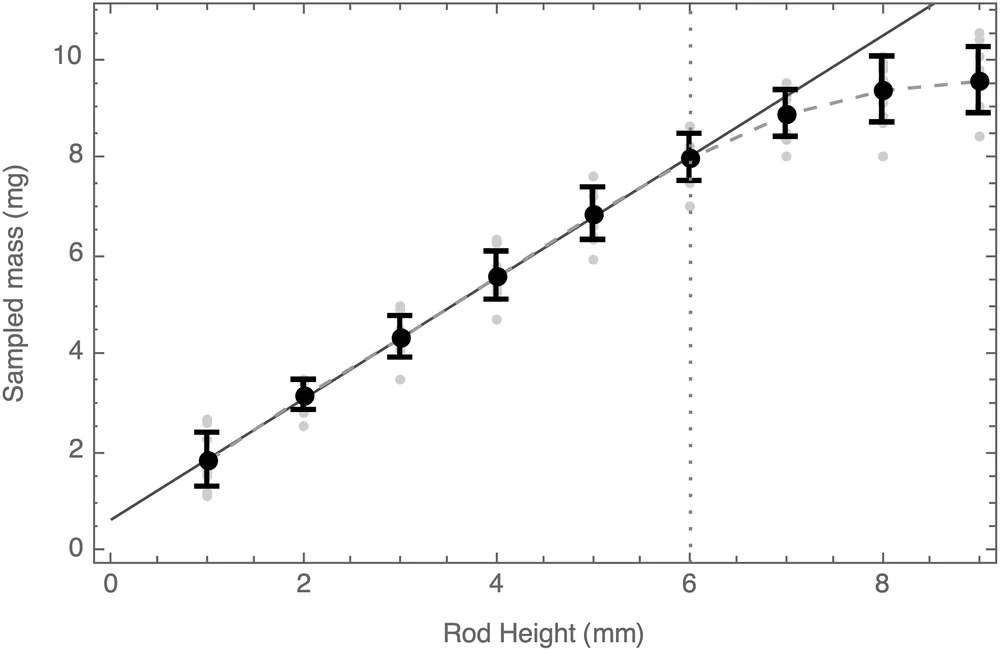
Calibration curve for Inhalac 70 using the 1.5 mm vacuum-assisted coring tool. Dispensed mass as a function of rod height. Gray points show individual measurements (*n* = 10 per height), and black circles indicate mean ± 1σ. The dashed line represents the linear regression over the 1–6 mm interval (R² = 0.9997). Deviation from linearity above 6 mm (vertical dotted line) defines the upper limit of the linear operating range (SP_lim_ ; m_max_). Within the linear domain, dispersion remains constant, enabling single-point σ determination during calibration. (Data available in supplementary data D2).

In the milligram regime, sampling dispersion remains constant throughout the linear operating range. This was verified by repeated dispensing experiments (*n* = 10) at each rod height (Fig. 12). The invariance of σ within the linear domain enables efficient calibration: dispersion can be characterized at a single reference rod height, and only one measurement per volume setting is required to determine the upper limit of linearity (SP_lim_ ; m_max_). Figure 12 provides a detailed view of the 2 mm dataset for Inhalac 70. A deviation from linearity is observed above 6 mm (gray vertical dotted line), while dispersion remains stable, as indicated by ±1σ around the mean dispensed mass. Linear regression across the 1–6 mm interval confirms the linear response (R² = 0.9997).

Because precision is determined after completion of the dispensing step through final weighing, it is governed entirely by the readability and repeatability of the analytical balance.

##### Speed and throughput

Throughput in STORMS is defined by workflow architecture rather than raw dispensing speed. By decoupling solid sampling from reaction plate population through pre-calibration, τ-tolerant chemical-space sampling, encapsulation and algorithm-driven matching, the platform removes feedback-controlled solid dispensing from the critical path of reaction preparation.

Compared with deterministic dispensing (~2 min per solid per well and strongly dependent on the material being handled), stochastic sampling reduces the handling time to 20–30 s per capsule, corresponding to a four- to fivefold increase in throughput. More importantly, the operation time of STORMS is largely independent of powder properties and depends primarily on robotic motion parameters. As a result, the time required for a sampling campaign scales predictably with the number of capsules to be generated rather than with the characteristics of the material being processed. Because sampling campaigns can be executed independently and in advance, reaction plate population becomes the rate-limiting step in closed-loop optimization workflows.

For a 96-well plate (e.g., Analytica Paradox^©^) with three solids per well, deterministic preparation requires 9.6 h. In contrast, stochastic population reduces preparation time to 2.4 h, and can be further decreased to ~0.8 h with high-speed pick-and-place robotics and optimized motion planning. This architecture enables continuous 24-h closed-loop optimization independent of operator intervention. Although encapsulation could be implemented in deterministic workflows, such systems would not benefit from reduced feedback adaptation and would remain constrained to single-iteration operation in the absence of a pre-generated capsule library and matching algorithm.

To be noted that the required calibration step introduces an initial time investment that must be taken into account into the overall evaluation; however, this cost is limited by the reduced number of required calibration points (ca. 5) and the invariance of dispersion within the linear domain. Importantly, calibration samples are not discarded but incorporated into the capsule library, minimizing material loss.

##### Powder compatibility domain

STORMS remains inherently material-dependent, particularly in the sub-milligram regime where fixed balance uncertainty and powder-specific properties contribute to relative error. The primary objective of STORMS is therefore not universal material flexibility, but rather the acceleration of sampling workflows and the architectural decoupling of sampling from reaction execution, thereby enabling efficient closed-loop optimization. In terms of powder compatibility, STORMS exhibits performance comparable to that of existing coring-based sampling systems. The application of vacuum at the top of the coring capillary slightly extends the operational domain by promoting powder filling within the capillary, particularly for materials with larger particle sizes and higher Carr indices (i.e., greater compressibility).

To more precisely assess the operational domain of the system in terms of powder type and achievable precision, complementary experiments were conducted using a range of representative materials. In general, sampling precision was found to be largely independent of powder properties. Instead, it is primarily governed by the characteristics of the weighing cell (repeatability and accuracy) and the stability of the surrounding environment, including mechanical vibrations, airflow fluctuations, and electrostatic disturbances. Powder properties mainly influence the ability of a material to be sampled successfully and the resulting width of the mass distribution.

Regarding sampling feasibility, a pronounced threshold was observed in passive mode (without vacuum assistance) at a Carr index of approximately 30. Below this threshold, the sampling success rate was close to 96% for powders with particle sizes smaller than the internal diameter of the sampling capillary. For powders with Carr indices above 30, the success rate decreased to approximately 6%. Particle size also plays a critical role, particularly for hard granular materials, such as NaCl, CaCl₂, and related salts. In these cases, the particle diameter must remain smaller than the internal diameter of the coring capillary to ensure reliable sampling.

A further limitation arises with highly cohesive materials. In such cases, the powder surface does not readily reform after sampling, leading to the formation of a cavity at the coring location and a progressive reduction in sampling performance. To mitigate this effect, vial-shaking strategies are currently being investigated to promote surface re-levelling between successive sampling operations. Additional studies are underway to further characterize the operational limits of STORMS across a broader range of chemically relevant materials and experimental conditions.

##### Further considerations

The stochastic–encapsulation strategy also carries the risk of generating unused capsules if experimental designs change. While material can ultimately be recovered, the approach is best suited to regularly used or strategically selected compound libraries.

Operational failure modes are limited during sampling because each capsule undergoes post-dispensing weighing, ensuring mass verification. During reaction plate population, incomplete capsule crushing represents the primary risk. Integration of force-feedback monitoring at the end-effector and potential vision-based verification are under development to further increase reliability.

## Discussion

STORMS shifts solid dispensing from a deterministic mass-targeting strategy to a stochastic, composition-centric paradigm. Rather than iteratively correcting toward a nominal value, the method accepts controlled deviations within predefined tolerances (τ) and restores compositional accuracy through downstream liquid adjustment. In this context, stochasticity is not a limitation but a design principle: statistical characterization of dispersion (σ) and bounded chemical-space tolerance are integrated into the workflow to enable speed, robustness, and architectural decoupling.

This reconfiguration separates sampling from reaction execution, removing feedback-controlled dispensing from the critical path and enabling parallelized, autonomous closed-loop experimentation. For high-throughput optimization, where structured exploration of chemical space is more important than exact mass matching, this approach provides a practical and scalable alternative to conventional deterministic methods.

The framework is inherently extensible. Provided dispersion can be quantified, the strategy can be translated to other mass regimes, solid-handling geometries, or material classes. Coupling stochastic dispensing with inline analytics and adaptive experimental design would further integrate sampling, reaction, and analysis into a continuous data-driven loop. STORMS thus defines a generalizable methodology for statistically controlled solid handling in autonomous chemical discovery.

## Methods

### Hardware

#### Robotic system

A UR3e collaborative robot (Universal Robots, Denmark) was used for all automated handling steps. The system provides a payload of 3 kg, a reach of 500 mm, and a repeatability of ±0.03 mm, which was sufficient for the present application. Its six degrees of freedom enabled operation within the confined geometry of the glovebox, while integrated force/torque sensing was used to detect contact with fragile components and the bottom of the vials.

The robot was equipped with a Robotiq Hand-E gripper. The gripper provides a 50 mm stroke, an adjustable grip force in the range of 20–185 N, and a repeatability of approximately 0.025 mm. Interchangeable fingertips were used to handle different components.

#### Powders selection

To assess the performance of the vacuum-assisted solid coring module across materials with defined physical characteristics, lactose powders with known properties were selected. Considered parameters included particle size distribution, bulk density, tapped density, and Carr’s index (Table 1). Flowability and compactability were also considered; however, because these properties were difficult to assess objectively in the present study, they were assigned only qualitative ratings.

#### Vials

Meggle powders were supplied in vacuum-sealed packaging and transferred into 4 mL glass vials (15 × 45 mm) for the experiments. To limit moisture uptake, vials were opened only during sample collection. In all experiments, powder fill heights were maintained between 10 and 30 mm.

#### Weighing system

Because the target sample masses extended down to 0.1 mg, high weighing precision was required. Spatial constraints within the glovebox additionally favored a compact instrument. A Mettler Toledo SPC215-111 weighing system was therefore selected. This provides a maximum capacity of 21 g, a readability of 0.01 mg, and a repeatability (σ) of 0.01 mg, enabling accurate quantification of small powder samples.

#### Glass capsules selection

The receiving glass capsules were selected to satisfy several constraints. They required sufficient internal volume to accommodate approximately 0.1–10 mg of powder, while their inner diameter had to be large enough to accept a 2.0 mm outer-diameter tube. At the same time, a clearance between the tube and capsule wall was necessary to reduce powder loss and to compensate for the effects of robotic positioning errors.

Wall thickness also required optimization. Excessively thick walls would complicate subsequent breakage, whereas excessively thin walls would increase the risk of mechanical failure during handling and provide insufficient molten material during laser sealing. Based on these criteria, capsules with outer diameter 2.8 mm, inner diameter 2.5 mm, and length 14 mm were selected.

#### Tubes and rods selection

Two interchangeable tube-and-rod sets were used in the vacuum-assisted solid coring module. The first consisted of a tube with outer diameter 1.8 mm, inner diameter 1.5 mm, and length 85 mm, paired with a rod of diameter 1.6 mm and length 100 mm. The second consisted of a tube with outer diameter 0.9 mm, inner diameter 0.6 mm, and length 80 mm, paired with a rod of diameter 0.6 mm and length 100 mm. All components were manufactured from 316 L stainless steel.

#### Contamination control

Cross-contamination was a central consideration throughout development and testing. To minimize carryover, the vacuum-assisted solid coring module incorporated replaceable tubes and rods fabricated from chemically inert stainless steel. The module itself was configured as a stationary tool to avoid unnecessary movement and vibration while powder was present in the chamber.

Robot motions and other manipulations within the glovebox were further restricted to minimize local air disturbance and vibration around the module. These measures were implemented to preserve sample integrity and reduce unintended powder transfer.

#### Vacuum-assisted solid coring module design

The vacuum-assisted solid coring module *(patent pending)* consists of two pneumatic grippers: a fixed gripper holding the outer steel tube and a movable gripper holding the inner rod. The movable gripper provides 0–10 mm travel with a positioning resolution of 0.1 mm, enabling precise adjustment of chamber height. The gripping interfaces are made from a specialized silicone that provides secure holding while preventing deformation of the thin-walled tube components.

#### Data acquisition

A dedicated workflow was developed for the purpose of quantifying the mass of powder collected by the vacuum-assisted solid coring module. All repetitive operations were performed by a programmed UR3e robotic arm (Universal Robots) in order to ensure precision and reproducibility. Lactose powders supplied by Meggle were transferred into 4 mL glass vials and prepared at fill heights of 10–30 mm. The sample mass was measured using a Mettler Toledo SPC215-111 weighing system.

The robot and balance communicate with a PC via Ethernet TCP/IP. In each experiment, the robot first retrieved an empty glass capsule from storage and placed it on the balance. Subsequent to a stabilization period of two seconds, the initial mass was recorded and transmitted to the PC. The vacuum-assisted solid coring module was then used to mount one of two interchangeable tube-and-rod assemblies (outer diameter 2.0 or 1.0 mm). Subsequently, the robot retrieved a powder-filled vial and positioned it beneath the sampling tube. The height of the chamber was predefined.

**Fig. 13:**
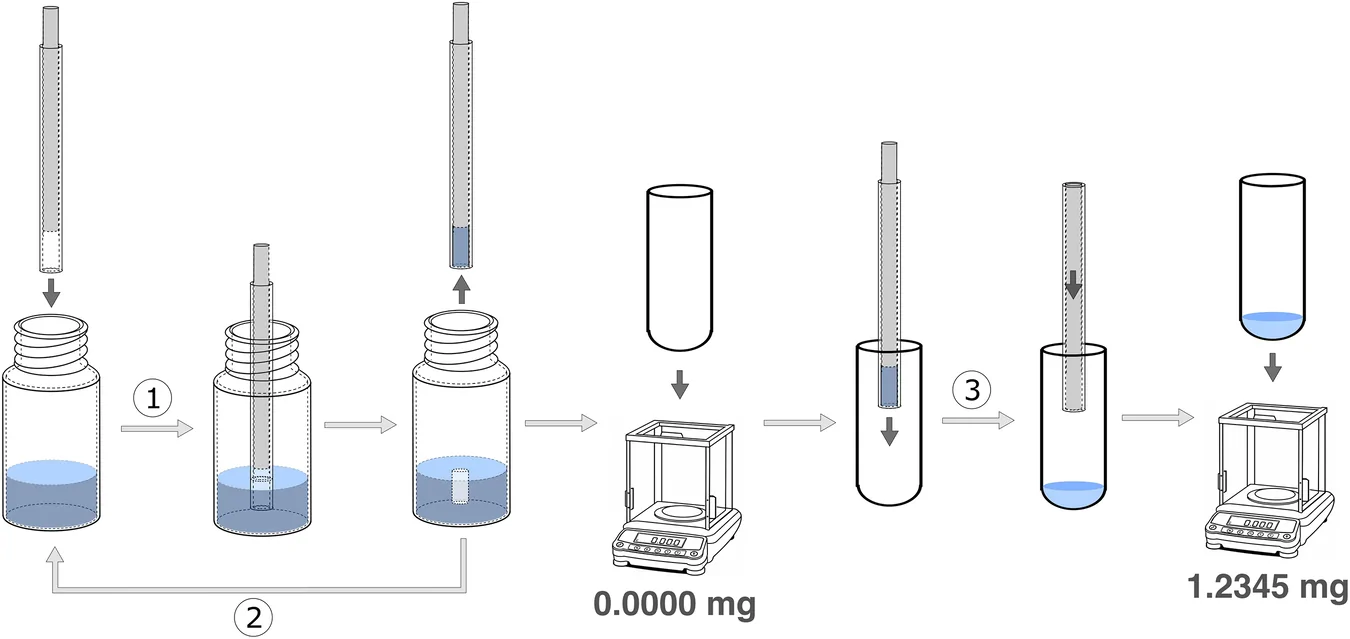
Data acquisition workflow. The initial step (1) in the process is the coring step. The tube-and-rod assembly is immersed in the vial containing the powder to be sampled, and the powder is extracted with the powder trapped in the chamber defined by the position of the rod. In the second step (2) of the procedure, the vial is subjected to a process of shaking with the objective of achieving the removal of the coring hole. Subsequently, the capsule recipient is tared, and in step 3, the cored powder is dispensed into the capsule by means of pushing the rod down. The final step is the weighing of the filled capsule.

The robot inserted the tube vertically into the powder bed and continued until contact with the vial bottom was detected by the robot’s force sensor. The vial was then withdrawn and returned to storage. Subsequently, the robot positioned the pre-weighed capsule beneath the tube outlet. The powder was dispensed by advancing the inner rod, thereby emptying the chamber. The capsule was then returned to the balance for a second weighing. The sampled powder mass was calculated as the difference between the final and initial capsule masses. The detailed workflow is described in Fig. 13 below.

#### Data handling

For each powder, the collected mass was recorded as a function of powder type, powder level in the vial, and chamber height. Raw data were processed using a custom-written Python script and exported as .csv files for further analysis.

#### Laser sealing system

Capsule sealing is performed using an air-cooled CO₂ working laser (max 100 W output power) with integrated red diode alignment laser (630–680 nm, <5 mW). The system comprises two physically separated enclosures: a laser box housing, the laser source and beam conditioning optics, and a sealing box into which the glass capsule is introduced for processing. This separation isolates the high-power optical components from the working environment and enables safe operation in standard glovebox conditions.

A filled glass capsule is transferred to the capsule holder by the robotic arm. The capsule holder is then lowered vertically into the sealing box. Once positioned, a PMMA window closes the sealing box opening, providing optical access for process monitoring while confining any stray radiation. A laser transfer line guides the beam from the laser box into the sealed enclosure, where it is directed onto the target zone of the glass capillary. The CO₂ wavelength is efficiently absorbed by the borosilicate glass, producing localized melting and fusion of the tube walls. During sealing, the capsule is rotated at 5 Hz to ensure circumferentially uniform glass flow and hermetic closure, while keeping the solid charge below the sealing zone out of thermal contact with the melt front.

The sealing process is monitored in real time by an integrated camera, which verifies successful closure before the pin holder retracts and the sealed capsule is returned for storage. This closed-loop visual verification step enables inline quality control without operator intervention and allows incomplete seals to be flagged automatically.

#### Vacuum system

The vacuum system uses a rotary vane vacuum pump, protected by a 5 µm in-line filter, allowing a vacuum level up to 0.1 mbar absolute to be achieved. A proportional pressure regulator is then used to adjust the vacuum level as required.

#### Experiment control

Experiment control was implemented using custom-made control software implemented in Python. The software coordinated TCP/IP communication between the PC, the Mettler Toledo weighing system, and the UR3e robot.

Balance communication was handled through a dedicated Python library based on MT-SICS interface commands (*github.com/janelia-python/mettler_toledo_device_python)*, whereas robotic commands were handled via Remote Procedure Calling in URScript. Measurement data were processed in Python and exported as .csv files for subsequent analysis.

### Software

2 sub systems have been developed for this architecture: the calibration and dispensing tool software (*github.com/swisscatplus/DC_Sampler*), and the matching algorithm (see supporting information).

The calibration and dispensing tool software is a Python-based tool that has been developed to control and automate the dispensing system using calibration data. The system stores data, including products, measurements, and model coefficients, in a SQLite database. During calibration, the system dispenses multiple volumes (1–9 mg), repeats the process to reduce noise and uncertainty, and uses the averaged results to identify a near-linear range. Then, it fits a polynomial model that links volume to delivered mass and saves the coefficients in the database. The software automatically identifies the deviation from linearity limit using the coefficient of determination (R²), which measures how well the data fits a linear model. By progressively adding data points and monitoring the R², the software detects when the alignment drops below a threshold (e.g., 0.97 in our case), which marks the end of the linear range. Sampling beyond this point becomes unpredictable and should be avoided or split into smaller doses.

For actual sampling, the chemist provides a target mass. The system then splits the requested mass into multiple capsules, if needed. It then uses the calibration model (by inverting the polynomial) to compute the required dispense volume and sends this volume to the hardware. The measured mass is then collected, and these steps are repeated until the target mass is reached within the desired tolerance.

Considering the matching algorithm, it is designed to assign microcapsules to chemical recipes. Its primary objective is to maximize the number of successful recipe completions. It operates by reading recipe batches and storage inventories both from Excel files. It translates each recipe’s target quantities into acceptable ranges, and organizes the optimization by product.

For each product, the system formulates a mixed-integer optimization model using Google OR-Tools (9.11.4210) and the SCIP solver (9.0.0)^43^. The optimization prioritizes meeting recipe tolerances while minimizing the number of vials used. It also ensures that stock and reactor capacity constraints are respected. If no feasible solution can be found, the software identifies and excludes problematic recipes. The final output provides a mapping of vial assignments and delivered quantities, as well as recipes that could not be matched with the available inventory.

## Supplementary information

Supplementary material STORMS

Description of Additional Supplementary Files

Supplementary Data

## Data Availability

All data supporting the findings of this study are provided within the paper and its Supplementary Data file. No human participants were involved, and no human data were collected in this study.

## Code availability

All codes and in-house developed software used for calibration, dispensing control, and matching algorithms are available through the Swiss Cat+ GitHub repository (https://github.com/swisscatplus) and in the Supplementary material file.
