# Supplementary material for: High-throughput solid microsampling through stochastic robotic automation
Source: Commun Chem. 2026 Jul 31;9:304. doi: 10.1038/s42004-026-02153-w (PMC13601550; doi:10.1038/s42004-026-02153-w)
Supplement: Supplementary file 1 [file 42004_2026_2153_MOESM1_ESM.pdf]

Supplementary material for

High-throughput solid microsampling through stochastic robotic automation

Adam Lisowski, Henryk Żołnowski, Keyan Villat, Alec Schmidt, Edy Mariano, Jean-Charles Cousty, Jérôme Guérin, Ashley Stark, Frédéric Vasserot, Achim Ammon, David Gueller, Florian de Nanteuil, Jean-Jacques Schwartz, Mathias Cherbuin, Marc Kunze, Giuseppe Costanzo, Pascal Miéville*

*pascal.mieville@epfl.ch

codes available at www.github.com/*swisscatplus/DC_Sampler*

S1 - Global STORMS workflow as deployed at EPFL Swiss Cat+ West Hub

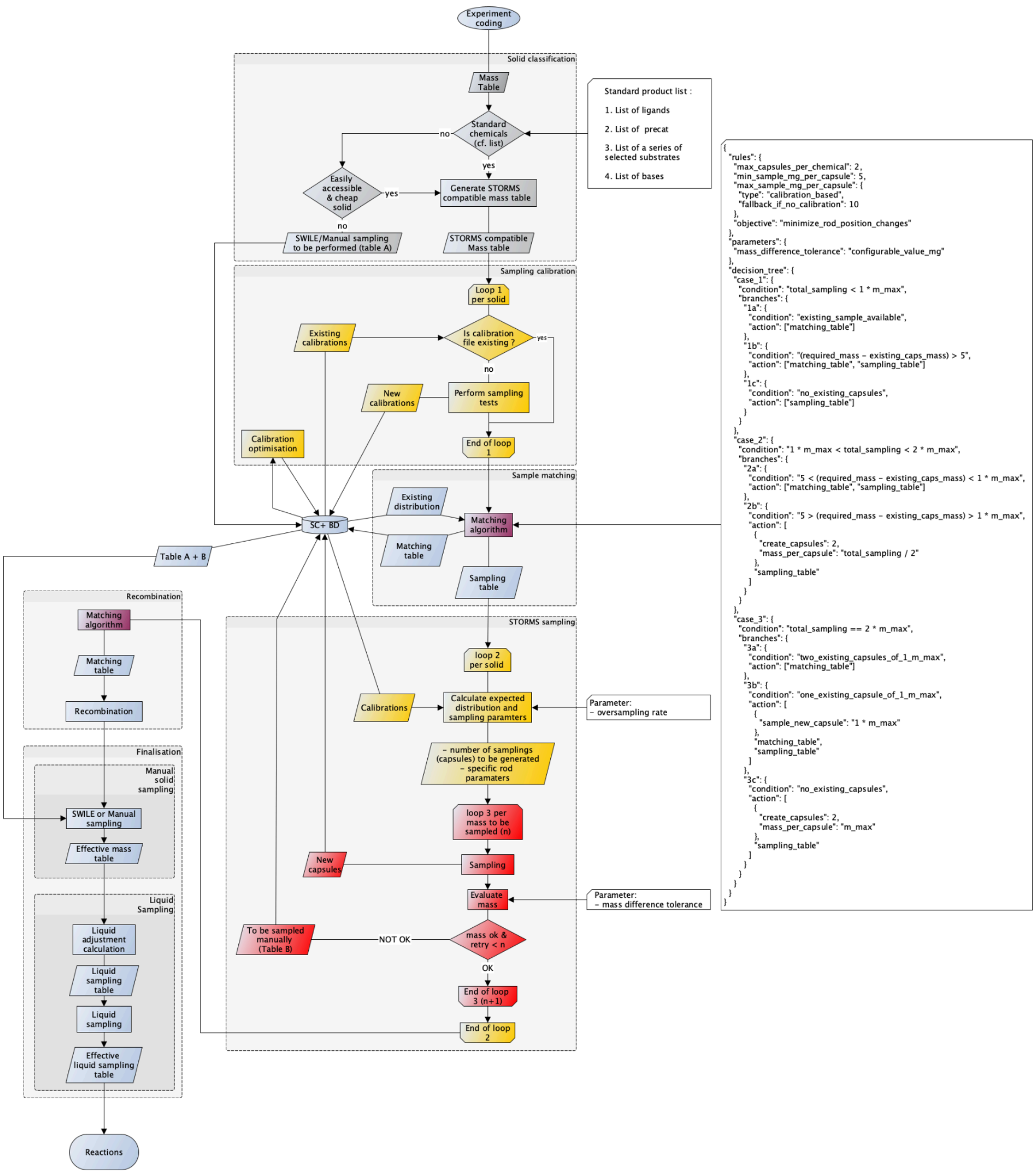

S2 - Rule-based decision algorithm containing- decision rules and parameters for the *Matching algorithm* as set at EPFL Swiss Cat+ West Hub

```
MASS MATCHING DECISION LOGIC

INPUT DEFINITIONS
- required_mass_mg
  Total target mass required for the chemical, expressed in mg.

- existing_sample_available
  Boolean variable indicating whether reusable sample material is available.

- existing_match_within_tolerance
   Boolean variable indicating whether at least one valid combination of existing capsules already matches the required mass within mass_difference_tolerance_mg.

- best_existing_match_mass_mg
  Mass in mg of the best reusable combination selected from existing capsules, with the constraint:
  best_existing_match_mass_mg < required_mass_mg

- existing_capsule_count
  Total number of reusable capsules currently available for the chemical.

- existing_capsules_of_m_max_count
  Number of reusable capsules currently available with mass exactly equal to m_max_mg.

- m_max_mg
  Maximum allowable mass per capsule, expressed in mg.

- mass_difference_tolerance_mg
  Acceptable tolerance on the mass difference, expressed in mg.

DERIVED VALUES
- small_sampling_threshold_mg = 5
- min_capsule_mass_mg = 3
- max_capsules_per_chemical = 2
- max_total_mass_mg = 2 * m_max_mg
- no_existing_capsules = (existing_capsule_count = 0)
- one_existing_capsule_of_m_max = (existing_capsules_of_m_max_count = 1)
- two_existing_capsules_of_m_max = (existing_capsules_of_m_max_count >= 2)
- best_existing_match_difference_mg = ABS(required_mass_mg - best_existing_match_mass_mg)
- residual_mass_to_sample_mg = required_mass_mg - best_existing_match_mass_mg
- required_capsule_count =
    IF required_mass_mg <= m_max_mg THEN 1
    ELSE 2

RULES
- all masses are expressed in mg
- maximum capsules per chemical = 2
- minimum sample mass per capsule = 3 mg
- maximum sample mass per capsule = calibration-based m_max_mg
- if no calibration exists, use 10 mg as m_max_mg
- reuse existing capsules whenever possible before creating new capsules
- objective = minimize rod position changes
- existing_match_within_tolerance = true means at least one valid combination of existing capsules satisfies:
  ABS(combination_mass_mg - required_mass_mg) <= mass_difference_tolerance_mg
- best_existing_match_mass_mg must always satisfy:
  best_existing_match_mass_mg < required_mass_mg
- residual_mass_to_sample_mg must always satisfy:
  residual_mass_to_sample_mg > 0
- if residual_mass_to_sample_mg > 0, a sampling_table must always be returned
- small_sampling_threshold_mg only determines whether the missing mass is sampled as a small adjustment or as a standard new capsule decision branch
- no branch may return matching_table alone if residual_mass_to_sample_mg > 0

OUTPUT DEFINITIONS
- matching_table
   Table containing the selected existing capsules or reusable sample combinations used to satisfy all or part of the required mass.

- sampling_table
  Table containing the quantities in mg that must be newly sampled to complete the required mass combination.

- create_capsules
  Number of new capsules that must be created.

- mass_per_capsule_mg
```

  Target mass in mg for each newly created capsule when multiple capsules of equal mass are created.

- sample_new_capsule_mg
  Target mass in mg for one newly created capsule when exactly one specific capsule mass must be sampled.

FUNCTIONAL LOGIC

```
IF required_mass_mg <= 0 THEN
  RETURN error
END IF
IF required_mass_mg > max_total_mass_mg THEN
  RETURN error
END IF
IF existing_match_within_tolerance = true THEN
  RETURN {
    matching_table,
    sampling_table: null,
    create_capsules: 0,
    mass_per_capsule_mg: null,
    sample_new_capsule_mg: null
  }
END IF
IF required_mass_mg <= m_max_mg THEN
  IF no_existing_capsules = true THEN
    RETURN {
      matching_table: null,
      sampling_table,
      create_capsules: 1,
      mass_per_capsule_mg: required_mass_mg,
      sample_new_capsule_mg: required_mass_mg
    }
  END IF
  IF existing_sample_available = true THEN
    IF residual_mass_to_sample_mg <= small_sampling_threshold_mg THEN
      RETURN {
        matching_table,
        sampling_table,
        create_capsules: 1,
        mass_per_capsule_mg: residual_mass_to_sample_mg,
        sample_new_capsule_mg: residual_mass_to_sample_mg
      }
    END IF
    IF residual_mass_to_sample_mg > small_sampling_threshold_mg THEN
      RETURN {
        matching_table,
        sampling_table,
        create_capsules: 1,
        mass_per_capsule_mg: residual_mass_to_sample_mg,
        sample_new_capsule_mg: residual_mass_to_sample_mg
      }
    END IF
  END IF
END IF
IF m_max_mg < required_mass_mg AND required_mass_mg < 2 * m_max_mg THEN
  IF no_existing_capsules = true THEN
    RETURN {
      matching_table: null,
      sampling_table,
      create_capsules: 2,
      mass_per_capsule_mg: required_mass_mg / 2,
      sample_new_capsule_mg: null
    }
  END IF
  IF residual_mass_to_sample_mg < m_max_mg THEN
    RETURN {
      matching_table,
      sampling_table,
      create_capsules: 1,
      mass_per_capsule_mg: residual_mass_to_sample_mg,
      sample_new_capsule_mg: residual_mass_to_sample_mg
    }
  END IF
  IF residual_mass_to_sample_mg >= m_max_mg THEN
    RETURN {
      matching_table,
      sampling_table,
      create_capsules: 2,
      mass_per_capsule_mg: required_mass_mg / 2,
      sample_new_capsule_mg: null
    }
  END IF
END IF
IF required_mass_mg = 2 * m_max_mg THEN
  IF two_existing_capsules_of_m_max = true THEN
    RETURN {
      matching_table,
      sampling_table: null,
      create_capsules: 0,
```

```
      mass_per_capsule_mg: null,
      sample_new_capsule_mg: null
    }
  END IF

  IF one_existing_capsule_of_m_max = true THEN
    RETURN {
      matching_table,
      sampling_table,
      create_capsules: 1,
      mass_per_capsule_mg: m_max_mg,
      sample_new_capsule_mg: m_max_mg
    }
  END IF
  IF no_existing_capsules = true THEN
    RETURN {
      matching_table: null,
      sampling_table,
      create_capsules: 2,
      mass_per_capsule_mg: m_max_mg,
      sample_new_capsule_mg: null
    }
  END IF
  RETURN {
    matching_table,
    sampling_table,
    create_capsules: 2,
    mass_per_capsule_mg: m_max_mg,
    sample_new_capsule_mg: null
  }
END IF
```
