# Supplementary material for: High-throughput solid microsampling through stochastic robotic automation
Source: Commun Chem. 2026 Jul 31;9:304. doi: 10.1038/s42004-026-02153-w (PMC13601550; doi:10.1038/s42004-026-02153-w)
Supplement: Supplementary file 2 [file 42004_2026_2153_MOESM2_ESM.pdf]

Description of Additional Supplementary Files:

File: Supplementary Data

Description:

D1 - Validation data for 1mm rod - masses from 0.1 to 1 mg

D2 - Validation data for 2mm rod - masses from 1 to 10 mg
