# Supplementary material for: High-throughput solid microsampling through stochastic robotic automation
Source: Commun Chem. 2026 Jul 31;9:304. doi: 10.1038/s42004-026-02153-w (PMC13601550; doi:10.1038/s42004-026-02153-w)
Supplement: Supplementary file 3 [file 42004_2026_2153_MOESM3_ESM.pdf]

Supplementary data for

High-throughput solid microsampling through stochastic robotic automation

Adam Lisowski, Henryk Żołnowski, Keyan Villat, Alec Schmidt, Edy Mariano, Jean-Charles Cousty, Jérôme Guérin, Ashley Stark, Frédéric Vasserot, Achim Ammon, David Gueller, Florian de Nanteuil, Jean-Jacques Schwartz, Mathias Cherbuin, Marc Kunze, Giuseppe Costanzo, Pascal Miéville*

*pascal.mieville@epfl.ch

D1 - Validation data for 1mm rod - masses from 0.1 to 1 mg

Rod height (mm)	1	2	3	4	5
Granulac 70	0.34	0.42	0.56	0.36	0.47
Granulac 200	0.35	0.57	0.56	0.54	0.62
Srbolac 400	0.34	0.64	0.74	0.90	0.90
Tablettose 70	0.43	0.53	0.35	0.72	0.64
Flowlac 100	0.27	0.52	0.79	0.89	1.16
Spherolac 100	0.30	0.68	0.82	0.75	0.99
Inhalac 70	0.30	0.54	0.70	0.61	0.39
Inhalac 230	0.40	0.65	0.73	0.79	0.93
Inhalac 500	0.31	0.37	0.35	0.43	0.35
Bicarbonate Na	0.66	1.04	1.02	1.33	1.07

D2 - Validation data for 2mm rod - masses from 1 to 10 mg

product_name	Rod height (mm)	mass (mg)
Bicarbonate Dec	1	0.80
Bicarbonate Dec	2	3.91
Bicarbonate Dec	3	5.18
Bicarbonate Dec	4	7.41
Bicarbonate Dec	5	9.95
Bicarbonate Dec	6	11.64
Bicarbonate Dec	7	14.29
Bicarbonate Dec	8	14.01
Bicarbonate Dec	9	12.94
FlowLac 100	1	0.83
FlowLac 100	1	3.15
FlowLac 100	2	1.89
FlowLac 100	2	5.50
FlowLac 100	3	3.10
FlowLac 100	3	7.52
FlowLac 100	4	4.39
FlowLac 100	4	9.28
FlowLac 100	5	5.93
FlowLac 100	5	11.45
FlowLac 100	6	7.03
FlowLac 100	6	13.66
FlowLac 100	7	8.27
FlowLac 100	7	15.40
FlowLac 100	8	9.45
FlowLac 100	8	15.95
FlowLac 100	9	10.80
FlowLac 100	9	15.77
FlowLac 100	9	8.47
GranuLac 200	1	0.98
GranuLac 200	2	2.69
GranuLac 200	3	4.22
GranuLac 200	4	5.70
GranuLac 200	5	7.13

**GranuLac 200**	6	7.67
**GranuLac 200**	7	8.53
**GranuLac 200**	8	8.59
**GranuLac 200**	9	9.15
**GranuLac 70**	1	1.02
**GranuLac 70**	2	2.85
**GranuLac 70**	3	4.32
**GranuLac 70**	4	5.99
**GranuLac 70**	5	7.85
**GranuLac 70**	6	8.83
**GranuLac 70**	7	10.50
**GranuLac 70**	8	11.17
**GranuLac 70**	9	11.33
**InhaLac 230**	1	0.89
**InhaLac 230**	2	2.47
**InhaLac 230**	3	3.74
**InhaLac 230**	4	5.26
**InhaLac 230**	5	7.24
**InhaLac 230**	6	8.47
**InhaLac 230**	7	10.13
**InhaLac 230**	8	11.19
**InhaLac 230**	9	13.12
**InhaLac 500**	1	0.01
**InhaLac 500**	2	0.02
**InhaLac 500**	3	0.05
**InhaLac 500**	4	2.32
**InhaLac 500**	5	2.70
**InhaLac 500**	6	2.41
**InhaLac 500**	7	4.35
**InhaLac 500**	8	2.34
**InhaLac 500**	9	3.39
**InhaLac 70**	1	1.14
**InhaLac 70**	1	2.72
**InhaLac 70**	1	2.65
**InhaLac 70**	1	1.63
**InhaLac 70**	1	1.2
**InhaLac 70**	1	1.81
**InhaLac 70**	1	1.97
**InhaLac 70**	1	1.72
**InhaLac 70**	1	2.29

**InhaLac 70**	1	1.52
**InhaLac 70**	2	2.58
**InhaLac 70**	2	3.45
**InhaLac 70**	2	3.06
**InhaLac 70**	2	3.07
**InhaLac 70**	2	3.44
**InhaLac 70**	2	3.53
**InhaLac 70**	2	3.48
**InhaLac 70**	2	3.22
**InhaLac 70**	2	2.86
**InhaLac 70**	2	3.12
**InhaLac 70**	3	3.49
**InhaLac 70**	3	4.36
**InhaLac 70**	3	4.99
**InhaLac 70**	3	4.28
**InhaLac 70**	3	4.43
**InhaLac 70**	3	4.91
**InhaLac 70**	3	4.51
**InhaLac 70**	3	4.13
**InhaLac 70**	3	4.11
**InhaLac 70**	3	4.38
**InhaLac 70**	4	4.7
**InhaLac 70**	4	5.42
**InhaLac 70**	4	5.72
**InhaLac 70**	4	5.56
**InhaLac 70**	4	5.83
**InhaLac 70**	4	5.58
**InhaLac 70**	4	5.28
**InhaLac 70**	4	6.36
**InhaLac 70**	4	6.29
**InhaLac 70**	4	5.31
**InhaLac 70**	5	5.95
**InhaLac 70**	5	7.64
**InhaLac 70**	5	6.66
**InhaLac 70**	5	6.43
**InhaLac 70**	5	6.33
**InhaLac 70**	5	7.24
**InhaLac 70**	5	6.73
**InhaLac 70**	5	7.38
**InhaLac 70**	5	7.26

**InhaLac 70**	6	7.03
**InhaLac 70**	6	8.08
**InhaLac 70**	6	7.89
**InhaLac 70**	6	8.19
**InhaLac 70**	6	7.54
**InhaLac 70**	6	8.27
**InhaLac 70**	6	8.27
**InhaLac 70**	6	8.12
**InhaLac 70**	6	8.68
**InhaLac 70**	7	8.36
**InhaLac 70**	7	8.86
**InhaLac 70**	7	9.2
**InhaLac 70**	7	8.98
**InhaLac 70**	7	9.55
**InhaLac 70**	7	9.29
**InhaLac 70**	7	9.39
**InhaLac 70**	7	8.08
**InhaLac 70**	7	8.76
**InhaLac 70**	7	8.53
**InhaLac 70**	8	8.72
**InhaLac 70**	8	9.9
**InhaLac 70**	8	10.06
**InhaLac 70**	8	9.63
**InhaLac 70**	8	9.92
**InhaLac 70**	8	9.81
**InhaLac 70**	8	9.79
**InhaLac 70**	8	9.13
**InhaLac 70**	8	8.89
**InhaLac 70**	8	8.02
**InhaLac 70**	9	9.65
**InhaLac 70**	9	10.56
**InhaLac 70**	9	10.43
**InhaLac 70**	9	10.07
**InhaLac 70**	9	9.79
**InhaLac 70**	9	9.65
**InhaLac 70**	9	9.09
**InhaLac 70**	9	9.08
**InhaLac 70**	9	8.97
**InhaLac 70**	9	8.47
**SorboLac 400**	1	0.98

**SorboLac 400**	2	2.59
**SorboLac 400**	3	4.09
**SorboLac 400**	4	5.50
**SorboLac 400**	5	6.34
**SorboLac 400**	6	6.37
**SorboLac 400**	7	6.51
**SorboLac 400**	8	6.45
**SorboLac 400**	9	9.35
**SpheroLac 100**	1	1.96
**SpheroLac 100**	1	0.93
**SpheroLac 100**	1	2.18
**SpheroLac 100**	1	1.44
**SpheroLac 100**	1	2.01
**SpheroLac 100**	2	3.23
**SpheroLac 100**	2	2.29
**SpheroLac 100**	2	3.09
**SpheroLac 100**	2	3.54
**SpheroLac 100**	2	3.01
**SpheroLac 100**	3	4.66
**SpheroLac 100**	3	3.79
**SpheroLac 100**	3	4.35
**SpheroLac 100**	3	5.02
**SpheroLac 100**	3	4.53
**SpheroLac 100**	4	6.44
**SpheroLac 100**	4	5.33
**SpheroLac 100**	4	6.04
**SpheroLac 100**	4	5.89
**SpheroLac 100**	4	6.12
**SpheroLac 100**	5	7.96
**SpheroLac 100**	5	6.94
**SpheroLac 100**	5	7.67
**SpheroLac 100**	5	7.96
**SpheroLac 100**	6	9.07
**SpheroLac 100**	6	8.20
**SpheroLac 100**	6	0.01
**SpheroLac 100**	6	8.59
**SpheroLac 100**	6	8.95
**SpheroLac 100**	7	9.63
**SpheroLac 100**	7	9.87
**SpheroLac 100**	7	10.47

**SpheroLac 100**	7	10.55
**SpheroLac 100**	7	10.33
**SpheroLac 100**	8	9.58
**SpheroLac 100**	8	10.95
**SpheroLac 100**	8	11.52
**SpheroLac 100**	8	11.43
**SpheroLac 100**	8	11.61
**SpheroLac 100**	9	9.52
**SpheroLac 100**	9	12.25
**SpheroLac 100**	9	12.13
**SpheroLac 100**	9	12.66
**SpheroLac 100**	9	12.58
**Tablettose 70**	1	1.17
**Tablettose 70**	2	2.44
**Tablettose 70**	3	3.42
**Tablettose 70**	4	4.54
**Tablettose 70**	5	5.70
**Tablettose 70**	6	6.81
**Tablettose 70**	7	7.87
**Tablettose 70**	8	9.04
